# Enteralgia1Read before the New York State Veterinary Medical Association, at New York City, September 6, 1895.

**Published:** 1895-11

**Authors:** L. E. Willyoung

**Affiliations:** Buffalo, N. Y.


### ENTERALGIA.[1]

By L. E. WILLYOUNG, D.V.S.,
BUFFALO, N. Y.

ENTERALGIA, signifying pain of the intestines, is commonly called colic when affecting horses. What I shall attempt to discuss before this audience is enteralgia or colic proper in the

[1] Read before the New York State Veterinary Medical Association, at New York City, September 6, 1895.

flatulent and spasmodic form, its many causes and different conditions, and its relation to the stomach, as we find it among our horses in every-day practice. Plenalvia proper pertains to ruminants mostly, although we deal with it frequently among horses. Plenalvia is virtually a mechanical gastric disorder, and we will consider it only under an acute indigestion, where we find a more gaseous distention than in plenalvia with ingesta.

Clinically speaking, it is a comfort to know or diagnose the true state of affairs you are to combat. While we all admit that time is precious in most cases, especially where the patient is so prone to get well before anything is administered, again others have been allowed to suffer and linger until death has almost relieved them; some are so violent in their contortions and struggles that it becomes dangerous to be hasty in your efforts to administer medicines. Among the physiological causes of enteralgia we find many elements which are important factors in its production.

First to be considered may be saliva, its chemical constitution, which contains its unorganized ferment ptyaline, and which is supposed to act only on starchy food during its digestion.

Second. Mastication depending on the teeth, and, as deglutition is partly involuntary, boluses of food being easily swallowed before properly masticated, defective teeth are conducive to gastric and intestinal disorders.

Third. Gastric secretions, the principal ferment pepsin, the reaction acid, HCl present to some extent, the quantity varying according to the stage of digestion, but is often found wanting, or may be in excess. The proteids should here be converted into peptones.

Fourth. Emotional causes often interrupt digestion in the human family, why should they not in the horse, as many horses exhibit abundant psychic phenomena? Here vomition would aid greatly in its relief, being controlled by the same medullary centre reflex act, but being mechanically obstructed by the anatomical conformation of the pendulous velum palati. This one barrier seems to be the most unfortunate defect in the anatomy of our noble soliped.

It is useless, physiologically, to torture you with intestinal digestion, but you will find causes leading to enteralgia in the intestines, among Peyer's patches, abundant in the ileum and the crypts of Lieberkuhn, as well as the emulsifying power of bile. Hence it may be said, anything which interferes with the proper salivation, mastication, digestion, and even assimilation of food is productive of enteralgia.

Our medical *confrères* no doubt find causes more numerous. But wait! how comparatively easy their diagnosis must be. The patient—man, woman, or child—has only to mention what was eaten, or taken with suicidal intent, and he receives his tablets, triturates, paregoric, or dilutions of the tenth or one hundredth potency. As Lowell says:

> "Too hasty to wait till art's ripe fruit should drop
> He must pelt down an unripe and colicky crop;"

hence the way of the transgressor.

Now let us return to colic as we find it in routine practice, where we have a condition of flatulency as the result of a somewhat hurried partial digestion; the food being churned, only partly chymified, in the great mash-tub or stomach, mixed imperfectly with its secretions. If by any of the causes above enumerated digestion is arrested, whether it be functional or otherwise, decomposition sets in; you have an enormous volume of gas evolved, filling the stomach, small intestine, colon, and cæcum. Some gas may be eliminated through the oral and nasal orifices, bringing with it some ingesta and liquids. Often nothing whatever is gotten rid of per anus, the colon and cæcum, being fully inflated, acting as a tampon to the posterior end, or rectum. In such cases paracentesis, or coloncentesis properly termed, should be performed at a point where the distention is greatest, whether on the right or left flank. Rectal injections or rectal manipulations with the hand and arm also prove valuable in some cases, for often this condition, if not speedily relieved, may lead to torsion of the intestine or rupture, especially if the animal rolls or is violent. Puncture of the colon, per rectum, has been advocated and tried, but my experience has been anything but pleasant in such attempts, for the reason that you are working in the dark, and the risk of producing some serious injury is far greater than the death of the animal from the mechanical distention.

Where the flatulency does not warrant the trocar, and you find dilatation and paresis of the intestine with no expulsion of gas, give eserine and pilocarpine, equal parts, the quantity of each depending on the size of the horse. 1½ to 3 grains of each will suffice for almost any animal when used hypodermically. The idiosyncrasy of the patient, the severity of the case, and the virtue of the drug have all to do in obtaining good results.

As we are aware, eserine stimulates involuntary muscular fibre while pilocarpine influences the sudorific, salivary, mesenteric, and nearly all secreting and excreting glands in the alimentary tract. Hence we have here one of the best speedy purgatives known. Its depressing cardiac influence is not to be lost sight of. For a period of from one to three hours following the use of eserine your patient usually suffers from a little cardiac weakness and involuntary muscular tremor, which can be benefited readily by the use of digitalis and carbonate of ammonia. Eserine is contraindicated in cases of acute indigestion where there is much gastric distention, as it will produce anti-peristalsis of the œsophagus as well as peristalsis proper of the intestines, and both do not seem to work together in harmony. As mentioned before the action of eserine is dependent entirely on the idiosyncrasy of the patient, and to be cautious, a minor dose had best be administered at the outset. We find the sulphate of eserine and the nitrate salt of pilocarpine best on account of their solubility in water.

There are many other remedies used in the treatment of flatulent colic, among which are turpentine, creosote, carbolic acid, sulphuric ether, chloroform, sulphate of magnesia, calcium carbonate, etc.

Spasmodic colic is the disease which prevails among horses so often confounded with a condition commonly termed by grooms, drivers, attendants, and owners as "The horse can't make his water; kidneys are out of order, etc." I believe, on the whole, that medical statistics would show that mankind is more predisposed to the disease "Can't make their water," in the manner in which Nature designed it to be made, than are horses. However, this misnomer has existed from time immemorial and will never die. For the ever-convincing proof to the attendant is the fact that as soon as an animal is relieved by the administration of anodyne remedies, whether or not diuretics are employed, the urine is freely voided, it is then that the groom becomes satisfied that his diagnosis was correct. In spasmodic colic we often find a retention of urine due in part to muscular constriction, and again colic may be confounded with paresis of the bladder due to a long retention and great quantity of urine contained. The causes of spasmodic colic may be enumerated as many. Intesitinal irritations due sometimes to superacidity of stomach, bilious colic, copious draughts of cold water, external exposure, producing chills, congestion of the bowels, toxic chemical irritants, calculi, and parasites. It is always well to know the cause producing colic, as very often when due to toxic influence antidotes are required. Often during spasmodic colic when an animal is writhing with pain so acute that the patient cannot remain on its feet long enough to take a drench, it behooves the medical attendant to administer remedies which will relieve the pain as speedily as possible. Chloral supersedes morphia in one respect: it will not produce constipation, while, on the other hand, it has acrid irritant properties if given largely. Combine the two, adding to it belladonna, Indian hemp, and an aromatic, you will find it to work admirably. This should be given in a concentrated form in gelatin capsules, first convincing the owner that you are not giving glass and all.

Torsion, or gut-tie of the intestine, is seen more frequently after severe attacks of spasmodic colic than following flatulency, and it is diagnosed with some difficulty. One of the chief leading symptoms of this condition is constant attempts at evacuation, the tail being elevated, and often a protruding rectum. I have had several cases during the past few years which I diagnosed as such, and the dejected countenance and anguished look were pitiful indeed. Unfortunately as yet no specific exists for the treatment or relief of this condition, and after all means are exhausted chloroform or ether and even venesection may prove beneficial. I believe more information can be obtained from one post-mortem than during the treatment of many such cases. Large doses of acetanilid work well in cases of a lingering nature, ounce doses of bromide of potash, equal parts by weight of camphor gum and carbolic acid, while sulphuric ether, laudanum and Jamaica ginger are still held as panacea among many. Homœopathy teaches us that the use of strychnia, arsenic, colocynth, and cocculus is indicated here, and for obstinate constipation opium produces never-failing results. If this be true, the oriental drug must be of questionable virtue, as often an interval of four or five days lapses before we have a free evacuation of the bowels following the use of it. Heroic measures, as severing the palatine artery, are resorted to by some, and while it hardly seems plausible that death from this hemorrhage would ensue, still such was the case in an animal which came under my observation during the present month.

During my remarks on colic I have omitted to mention toxic enteralgia, which really belongs to gastro-enteritis. Among the many causes may be mentioned caustic alkali, sulphuric acid, tartar emetic, arsenic, phosphorus, mercurials, lead, zinc, carbolic acid, cantharides, acrid oils, and many irritant vegetable poisons ingested while at pasture; also colic due to aneurisms; verminous colic; colic from intestinal parasites, calculi, etc., all of which require special treatment.

On the whole, this prevalent disorder is most frequently met with resulting from food changes following injudicious feeding, an unintentional abuse by a class of people existing in this rapidly rising generation, in which Darwin lived and started the ball of evolution. It is to be hoped that those who struggle for existence (even if it is not the fittest that survive) will endeavor to maintain and promote the good work begun by our Greek and Roman teachers. Have compassion on the speechless beasts, treat them humanely, bearing in mind that their sensitive systems possess in part a degree of sensation, pleasure, disgust, and pain, which require the same attention, sympathy, and treatment that man does, and receives more often than he deserves in return for the cruelty and abuse wrought upon this great and noble animal, the horse.

